# Growth kinetics of high-grade serous ovarian cancer: implications for early detection

**DOI:** 10.1038/s41416-025-03082-6

**Published:** 2025-06-12

**Authors:** Bharath Narayanan, Thomas Buddenkotte, Hayley Smith, Mitul Shah, Susan Freeman, David Hulse, Gabriel Funingana, Marie-Lyne Alcaraz, Mireia Crispin-Ortuzar, James Brenton, Paul Pharoah, Nora Pashayan

**Affiliations:** 1 Department of Public Health and Primary Care, University of Cambridge, Cambridge, UK; 2 Department of Nuclear Medicine and Interventional Radiology, University Hospital of Hamburg-Eppendorf, Hamburg, Germany; 3 Department of Oncology, University of Cambridge, Cambridge, UK; 4 Department of Radiology, Addenbrooke’s Hospital, Cambridge, UK; 5 Early Cancer Institute, University of Cambridge, Cambridge, UK; 6 Cedars Sinai Medical Center, Los Angeles, CA USA

**Keywords:** Ovarian cancer, Cancer screening, Metastasis

## Abstract

**Background:**

High-grade serous ovarian cancer (HGSOC) is the most lethal gynaecological cancer with patients routinely diagnosed at advanced stages. Evidence from randomized controlled trials indicates that annual screening may not reduce cancer-related deaths. We aim to characterise the growth kinetics of HGSOC to understand why early detection failed and under what conditions it might prove fruitful.

**Methods:**

We analysed data from 597 HGSOC patients and identified 34 cases with serial CT scans. We calculated the growth rates of lesions in the ovaries/pelvis and the omentum and estimated the time to metastasis using a Gompertz model. Finally, we simulated ultrasound and CA125 based screening in a virtual population of patients.

**Results:**

Growing lesions in the ovaries and the omentum doubled in volume every 2.2 months and 1.8 months respectively. The 11 cases with growing lesions in both sites had a median interval of 13.1 months between disease initiation and the onset of metastasis. Our simulations suggested that 27% of tumours would metastasise before screen detection. The remainder would provide a median window of 4.2 months for detection before metastasis.

**Conclusion:**

Our results suggest that HGSOC lesions have short times to metastasis, preventing effective early detection using current screening approaches.

## Introduction

Most patients with ovarian cancer are diagnosed at late stages with poor prognosis [1]; the 5-year survival rates of these individuals is less than half that of those diagnosed at early stages [2]. High-grade serous ovarian cancer (HGSOC) is the commonest histotype and is responsible for the majority of advanced-stage diagnoses and cancer-related deaths [3]. HGSOC originates in the fallopian tube as pre-cancerous serous tubal intraepithelial carcinoma (STIC) lesions [4]. They then detach from the fallopian tube and spread non-hematogenously to the ovaries, peritoneum, omentum and the lymph nodes [5] where the disease can grow to a large volume without causing symptoms. Early detection initiatives aim to reduce mortality by detecting HGSOC as early as possible. However, screening for ovarian cancer is a challenging task, as evidenced by the lack of mortality benefit in recent trials. The United Kingdom Collaborative Trial of Ovarian Cancer Screening (UKCTOCS) [6] found that annual screening using CA125 and ultrasound did not reduce cancer-related deaths over a median 16-year follow up period. Similarly, the Prostate, Lung, Colorectal and Ovarian cancer screening (PLCO) trial [7] in the United States found no meaningful reduction in mortality through ultrasound (US) based screening. Consequently, both trials recommended against introducing screening programmes for ovarian cancer.

These findings suggest that by the time HGSOC can be screen-detected, it is already too late for most patients, regardless of the stage at which they are diagnosed. The success of screening programmes is linked to the growth rate of lesions and the interval between tumour initiation and metastatic dissemination. The lethality of HGSOC makes it challenging to gather sufficient longitudinal measurements to characterise its growth kinetics. Researchers have thus used mathematical models to infer the growth kinetics using the little data that is available. Brown and Palmer used the volumes and stage distributions of occult cancers identified during prophylactic bilateral salping-oopherectomy to estimate the tumour volume doubling time and the time spent by tumours in early stages before progression [8]. Danesh et al. used the growth rates from Brown and Palmer to calculate the interval between the earliest possible screen detection using US and the initiation of metastases using a branching process model [9]. Havrilesky et al. took a different approach and fitted a two-phenotype multi-state model to the stage distribution and outcomes from individual case data to identify the time spent by indolent (type-I) and aggressive (type-II) ovarian cancers in stage I/stage II [10]. Recent studies have been tailored to the growth kinetics of HGSOC in particular. Botesteanu et al. estimated the doubling time of HGSOC using volumes of diseased ovaries from 9 patients at single time points along with assumptions about the size of a normal ovary. They then developed a stochastic Gompertz model of HGSOC to identify the interval between a tumour being detectable through US and it reaching a life-threatening size [11]. Bedia et al. used longitudinal CA125 data from 504 women in the UKCTOCS trial to infer the growth rate of those HGSOC tumours that secreted CA125 [12]. They also reported the preclinical detectable period (PCDP) between noticing a change in CA125 dynamics and eventual clinical diagnosis.

None of the aforementioned studies report the observed growth rates from two or more volume measurements. Although Bedia et al. used multiple biomarker measurements in their study, CA125 is a poor proxy for primary disease burden as we do not know the contribution from healthy tissue and each disease site. Additionally, Havrilesky et al. calculated the duration spent in early stages using staging/incidence data without information on the volume of metastatic disease. Therefore, these values are not true reflections of the time interval before metastasis as we do not know if the stage I/II cases in these datasets contained micro-metastases at diagnosis. In a similar vein, the PCDP reported by Bedia et al. does not tell us the window for detecting tumours before metastasis.

In this paper, we address the gaps in current knowledge by characterising the growth kinetics of lesions in the ovaries/pelvis and the omentum using longitudinal volumetric data from 34 HGSOC patients. The two disease sites were chosen as they account for most of the disease burden and are generally the most frequent locations for HGSOC [13]. Our work distinguishes itself from previous studies by reporting (i) the observed tumour growth rates between two volume measurements and (ii) the time interval between disease initiation and the onset of metastasis, using the growth kinetics of lesions in both disease sites.

## Methods

### Data

We used clinical data from a prospective cohort study, Cambridge Translational Cancer Research Ovarian Study 04 [14] (CTCR-OV04), designed to investigate the mechanisms of treatment response in ovarian cancer. We obtained access to individual data from 597 women in this cohort with histologically confirmed HGSOC, enroled between September 2010 and September 2022. The patients, aged 66.9 ± 10.6 years were predominantly white (98.3%) with stage III/IV disease (85.6%) (Table 1).

**Table 1:** Characteristics of all 597 HGSOC patients and the 34 patients with a growing lesion in either the ovaries/pelvis or the omentum.

	Volume analysis (*N* = 34)	All HGSOC (*N* = 597)
Age (yrs)	68.8 ± 10.5	66.9 ± 10.6
Stage		
I	1 (3.0)	49 (8.9)
II	0 (0.0)	30 (5.5)
III	26 (78.8)	308 (56.2)
IV	6 (18.2)	161 (29.4)
NA	1	49
gBRCA status		
Wildtype	21 (95.4)	261 (83.1)
1/2/m	1 (4.6)	40 (12.7)
VUS	0	13 (4.1)
NA	12	283
Family history of cancer		
Breast	3 (8.8)	40 (6.7)
Ovarian	3 (8.8)	26 (4.7)
Other	9 (26.5)	87 (14.6)
Ethnicity		
White	30 (96.8)	510 (98.3)
Asian	1 (3.2)	7 (1.3)
Other	–	2 (0.4)
NA	3	78

Percentages are shown in brackets. Age is reported as the mean ± SD. Percentages were calculated after excluding cases with no available information (NA). The same individual could have multiple family members with a history of cancer; therefore the numbers and percentages are not additive. Three individuals in the HGSOC dataset did not have reported ages

**Fig. 1:**
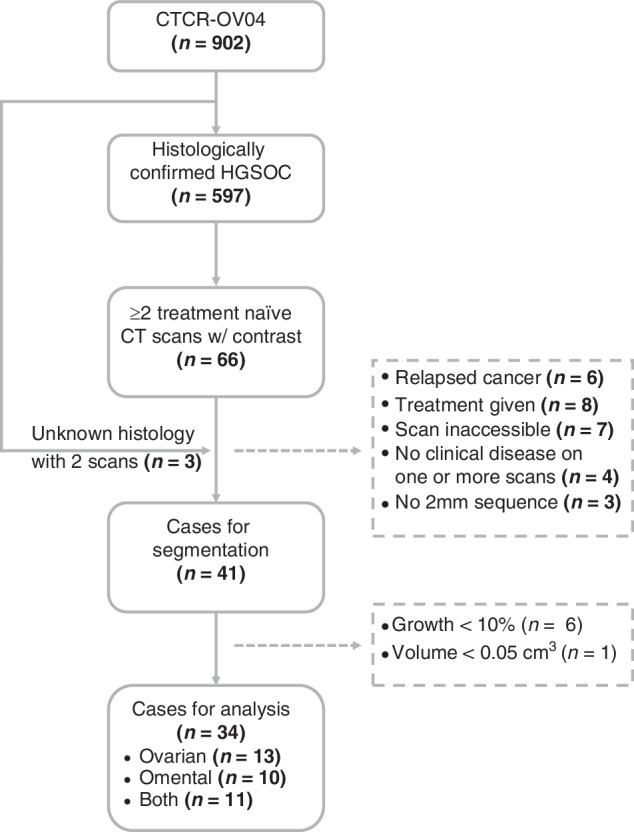
Consort diagram showing the cases for volumetric analysis. There were 34 cases that had two or more scans with clinically observed disease >0.05 cm^3^ and at least 10% growth in either the pelvis/ovaries or the omentum. Excluded cases are shown in the dashed boxes.

Sixty-six patients had multiple CT scans with contrast before treatment, making them suitable for modelling tumour growth kinetics. We included three more patients with multiple CT scans who had no confirmed histotype, resulting in a total of sixty-nine candidates for analysis. The reasons for multiple scans before treatment included re-staging to assess the extent of metastatic disease following a delay in the commencement of chemotherapy, confirmation of a suspicious lesion, or new symptoms that could be due to unobserved lesions in the first scan. We only considered those cases with at least two scans taken with contrast at a 2 mm slice thickness and clinically observed primary disease on both scans; forty-one cases satisfied these criteria (Fig. 1).

We used CT scans to evaluate the growth kinetics of HGSOC as they contain information on disease burden in multiple sites; this enabled us to estimate the time interval between the initiation of lesions in different sites.

### Scan segmentation

We applied a deep-learning algorithm called *ovseg* [15] on the CT scans to automatically segment lesions in the ovaries/pelvis and the omentum. *Ovseg* is a state-of-the-art segmentation framework that was designed and finetuned to segment lesions in HGSOC. It demonstrated performance comparable to a junior radiologist and achieved a Dice similarity coefficient of up to 71 +/− 12 for ovarian/pelvic lesions and 61 +/− 24 for omental lesions on external test data.

Although *ovseg* was trained on images acquired at a slice thickness of 5 mm, we used scans acquired at intervals of 2 mm as this provided a higher resolution and also expanded the number of suitable cases. Given this change in resolution, we revalidated the method by comparing the volumes obtained using the two slice thicknesses for 38 scans that had both resolutions available and a volume of at least 0.05 cm^3^.

### Volume calculation

We calculated the total tumour burden in each disease site from the binary segmentation mask outputted by *ovseg* using:

$${V}_{{ov}/{om}}=\mathop{\sum }\limits_{i=1}^{{N}_{v}}{z}_{i}^{{ov}/{om}}\cdot {dv}$$

Where ${N}_{v}$ is the total number of voxels in the scan sequence, ${z}_{i}$ is 1 if the voxel is part of a lesion and 0 otherwise, and ${dv}$ is the volume of a voxel.

### Doubling time calculation

We considered cases for doubling time calculations if they had at least 10% increase in volume between successive scans, and a minimum burden of 0.05 cm^3^ in either disease site; these requirements mitigated the chances of the results being skewed by artefacts.

We calculated the doubling time (TVDT) assuming an exponential growth model which provides the simplest way to capture the growth rate from two volume measurements.

1 $$\frac{1}{V}\frac{{dV}}{{dt}}=\alpha ;\alpha =\frac{1}{{dt}}{\mathrm{ln}}{V}_{2}/{V}_{1}$$

2 $${TVDT}=\frac{\mathrm{ln}2}{\alpha }$$

where *α* is the specific growth rate of the tumour, and *V*_2_ and *V*_1_ are the tumour volumes measured at a time interval of *dt*.

### Gompertz model of tumour growth

We sought a model that could best estimate the time of initiation of the lesions at each disease site (ovarian or omental) from the available volumetric data. The constant growth rate assumption of the exponential model (Eq. (1)) makes it unsuitable for this task as tumour growth has been shown to decrease with size until it becomes almost negligible at extremely large volumes [16–18]. Instead, we used a Gompertz model as it has been found to better mimic preclinical [18–20] and clinical [21] tumour growth patterns rate (Eq. (3)).

3 $$V\left(t\right)= 	 \,\,{V}_{0}\cdot \exp \left[K\left(1-{e}^{-\beta t}\right)\right] \\ \frac{1}{V}\frac{{dV}}{{dt}} = 	 \,\,\beta K{e}^{-\beta t}$$

*V*_0_ is the initial volume, assumed to be a single cell (10^−9^ cm^3^), *β* is the decay constant, and $K=\mathrm{ln}\frac{{V}_{\infty }}{{V}_{0}}$, where ${V}_{\infty }$ is the carrying capacity of the tumour. We set ${V}_{\infty }$ based on the maximal ovarian and omental volumes in our dataset.

The OV04 data consisted of two volume measurements, $({V}_{1},\,{V}_{2})$ along with the time interval, $\Delta t={t}_{2}-{t}_{1}$, between the two scans. The time interval, ${t}_{1}$, between initiation of the specific lesion and the first scan is unknown and thus needed to be estimated along with *β* (Eq. (4)).

4 $$V(\Delta t)={V}_{0}\cdot \exp \left[K\left(1-{e}^{-\beta \left({t}_{1}+\Delta t\right)}\right)\right]$$

Here, the volume V is a function of the time interval Δ*t*.

### Population modelling

We used a nonlinear mixed effects model (NLME) to fit the data and estimate the time between tumour initiation and the first volume measurement in the two disease sites; this population approach to modelling enabled us to estimate the overall trend in growth kinetics while also accounting for inter-subject variability.

NLME assumes that all individuals belong to a single population, and that their individual model parameters, ${{\boldsymbol{\theta }}}^{{\boldsymbol{i}}}$, are distributed normally around the population average, ${{\boldsymbol{\theta }}}^{{\boldsymbol{pop}}}$, with a variance matrix ***D*** (Eq. (6)). The observation, ${y}_{j}^{i}$, of individual *i* at time ${t}_{j}^{i}$; is a function of ${{\boldsymbol{\theta }}}^{{\boldsymbol{i}}}$ with an added error term, ${\epsilon }_{j}^{i}$, that follows a standard normal distribution and is scaled by *λ* (Eq. (5)).

5 $${y}_{j}^{i}=f\left({t}_{j}^{i};{{\boldsymbol{\theta }}}^{{\boldsymbol{i}}}\right)+{\lambda \cdot \epsilon }_{j}^{i}$$

6 $${{\boldsymbol{\theta }}}^{{\boldsymbol{i}}}= 	\,\, {{\boldsymbol{\theta }}}^{{\boldsymbol{pop}}}+N(0,\,{\boldsymbol{D}})\\ {\epsilon }_{j}^{i}= 	\,\, N(0,1)$$

Normally, ${y}_{j}^{i}$ and $f({t}_{j}^{i};{{\boldsymbol{\theta }}}^{{\boldsymbol{i}}})$ would represent the measured lesion volumes and the expression for Gompertzian growth (Eq. (4)), respectively. However, we reformulated the expressions for ${y}_{j}^{i}$ and $f({t}_{j}^{i};{{\boldsymbol{\theta }}}^{{\boldsymbol{i}}})$ to facilitate convergence and used log-transformed parameters to ensure strictly positive values for *β* and *t*_1_. (Eq. (7)).

7 $${y}_{j}^{i}= 	 -{\mathrm{ln}}\left(1-\frac{1}{K}{\mathrm{ln}}\frac{{V}_{\rm{j}}^{\rm{i}}}{{V}_{0}}\right)\\ f({\Delta t}_{j}^{i};{{\boldsymbol{\theta }}}^{{\boldsymbol{i}}})= 	\,\, {e}^{{\left(\mathrm{ln}\beta {t}_{1}\right)}^{i}}+{e}^{{\left(\mathrm{ln}\beta \right)}^{i}}\Delta {t}_{j}^{i}$$

Here, ${{\boldsymbol{\theta }}}^{{\boldsymbol{i}}}=\{{\left(\mathrm{ln} \, \beta \right)}^{i},\,{\left(\mathrm{ln} \, \beta {t}_{1}\right)}^{i}\}$, while *f* is a function of the time interval $\Delta {t}_{j}^{i}$. The first observation at ${t}_{1}^{i}$ for each patient corresponds to $\Delta {t}_{1}^{i}=0$. We estimated the parameters (${{\boldsymbol{\theta }}}^{{\boldsymbol{pop}}},{\boldsymbol{D}},$ and *λ*) by maximising a linearised approximation of the log likelihood [22] using the *nlmefit* package implemented in Matlab 2024 [23].

The NLME algorithm provides us with the fixed effects estimates of the log transformed parameters ${\left(\mathrm{ln}\beta \right)}^{{pop}}$ and ${\left(\mathrm{ln}\beta {t}_{1}\right)}^{{pop}}$; however, the variable of interest is the time since initiation of the ovarian or omental lesions, ${t}_{1}$. We calculated the population level ${t}_{1}^{{pop}}$ using the formula, ${t}_{1}^{{pop}}={e}^{{\left(\mathrm{ln}\beta {t}_{1}\right)}^{{pop}}-{\left(\mathrm{ln}\beta \right)}^{{pop}}}$. We then estimated the 95% CI of ${t}_{1}^{{pop}}$ from the standard errors of the transformed variables (Supplementary Note 1).

We compared the value of ${t}_{1}^{{pop}}$ estimated using Gompertzian growth against those from an NLME with exponential growth (Eq. (8)) where ${{\boldsymbol{\theta }}}^{{\boldsymbol{i}}}={\{\left({\mathrm{ln}} \, \alpha \right)^{i},{\mathrm{ln}}\left(\alpha {t}_{1}\right)^{i}\}}$.

8 $${y}_{j}^{i} = 	\,\, {\mathrm{ln}}\frac{{V}_{\rm{j}}^{\rm{i}}}{{V}_{0}} \\ f\left({{\Delta }{t}_{j}}^{i};{{\boldsymbol{\theta }}}^{{\boldsymbol{i}}}\right) = 	\,\, {e}^{{\left({\mathrm{ln}} \,{ \alpha }{t}_{1}\right)}^{i}}+{e}^{{\left({\mathrm{ln}} \,{ \alpha }\right)}^{i}}\Delta {{t}_{j}^{i}}$$

### Estimating the window-of-opportunity for screening in the OV04 dataset

**Fig. 2:**
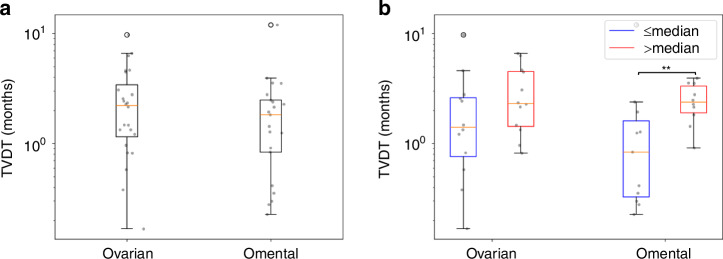
Tumour volume doubling times (TVDT) in HGSOC. TVDT **a** by tumour site (24 ovary/pelvis and 21 omentum) and **b** by tumour site and tumour volume at diagnosis - above (red) and below (blue) the median volume.

We used the individual parameter estimates, ${{\boldsymbol{\theta }}}^{{\boldsymbol{i}}},$ to calculate the ${t}_{1}^{i}$ for each of the 11 cases with growing lesions in both disease sites and identify the window of opportunity (WOO) for early detection; this is the interval between the earliest possible screen detection and the onset of metastasis. We estimated the time to metastasis (Fig. 2), ${\rm{TTM}}={t}_{1,p}^{i}-{t}_{1,s}^{i}$, given by the interval between the initiation of disease in the primary (p) and secondary (s) disease sites. The primary site was not always the ovaries/pelvis as four cases were estimated to have disease initiated in the omentum first; we retrospectively found that these were cases of peritoneal cancer which is also classified as HGSOC [24].

We then calculated the WOO for screening between the point at which the designated primary site reaches the US or CA125 detection thresholds of 0.5 cm^3^ and 0.015 cm^3^ respectively and the time of initiation of disease in the secondary site. These detection thresholds were informed by studies on the size distribution of image-detected tumours [25, 26] and a model-based estimate of the tumour burden at CA125 detection [27, 28]. Although we used CT scans for characterising the growth kinetics of the tumours in this study, we used the US detection threshold in our simulations as screening is most likely to be done by ultrasound.

### Estimating the window-of-opportunity for screening in an in-silico population

We simulated a virtual population of 10,000 patients to identify how many people could theoretically benefit from screening, and what the WOO would be in these individuals. For each case, we assigned the primary disease site with probabilities ${p}_{{om}}/{p}_{{ov}}$ and then sampled the Gompertz decay rate *β* from the corresponding distribution, while keeping ${V}_{\infty }^{{ov}/{om}}$ fixed. We used a value of 0.36 for *p*_*om*_ as 4 out of 11 cases in our cohort were expected to develop omental disease first; the results were agnostic to the choice of *p*_*om*_. Next, we sampled the size at which metastasis is seeded, *V*_*met*_, from the Gompertz estimate of the primary tumour at metastasis in the 11 patients with growing lesions in both sites. Finally, we calculated the time, *t*_*met*_, taken for the primary tumour to reach ${V}_{{met}}$ and the WOO between the primary tumour reaching the CA125/US detection limits and *t*_*met*_.

## Results

### Dataset

We segmented lesions for 41 patients with a new diagnosis of HGSOC who had evaluable CT imaging before neoadjuvant chemotherapy or surgical treatment. We compared the volumes calculated at 2 mm and 5 mm slice thicknesses for 38 scans across 20 patients; we observed a median difference of 3.3% (IQR: 1.6–8.4) and 3.8% (1.2–10.7) for the ovarian and omental lesions respectively. We calculated the TVDT for 34 out of the 41 cases that had growing lesions in at least one of the two disease sites (Table 1) - 13 had growing ovarian lesions only, 10 had growing omental lesions only, and 11 had growing lesions in both disease sites (Supplementary Note 2 and Supplementary Data 1).

These 34 cases had a greater proportion of stage III/IV diagnoses (97.0% vs 85.6%) and a higher mean age at diagnosis (68.8 vs 66.9) than the general HGSOC subpopulation (Table 1). The proportion of individuals who identified as ‘white’ in the volume analysis subset reflected their representation in the larger HGSOC dataset (96.8% vs 98.3%) while there was a decrease in the percentage of women with *BRCA* mutations of known significance (4.6% vs 12.7%). The median interval between scans was 31 days (IQR: 16.3–43.5).

### HGSOC lesions double in volume every two months

There were a total of 21 omental lesions and 24 ovarian/pelvic lesions that were amenable to TVDT calculations. The omental lesions grew faster than the omental/pelvic lesions (*p*-value 0.16) with a median TVDT of 1.8 months (IQR: [0.8, 2.5]) as compared to 2.2 months (IQR: [1.2, 3.4]) (Fig. 2a). In both disease sites, lesions smaller than the median volume grew faster than those that were larger (Fig. 2b); however, only the omental lesions displayed a statistically significant difference (*p*-value 0.007).

### Median time to metastasis is 13 months for cases with growing primary and metastatic lesions

The estimated population-level age of the tumour at first scan, ${t}_{1}^{{pop}}$, was 20.4 months months (95% CI [13.6, 30.6]) for ovarian/pelvic lesions and 14.7 months (95% CI [9.4, 22.9]) for omental lesions. These values were substantially lower than those calculated using an exponential growth assumption – 66.1 months (95% CI [44.5, 98.1]) for ovarian/pelvic lesions and 46.9 months (95% CI [29.6–74.3]) for omental lesions. This discrepancy is because the Gompertz model assumes that smaller tumours grow faster, leading to lower estimates of the age of a lesion. We found that these population estimates of ${t}_{1}^{{pop}}$ were robust to measurement error and the choice of the carrying capacity, ${V}_{\infty }$ (Supplementary Note 3).

**Fig. 3:**
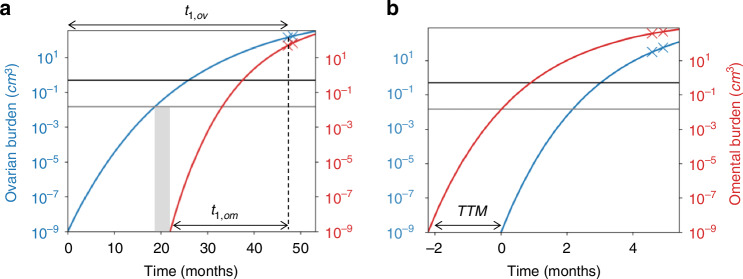
Gompertz growth profiles for two cases. **a** One that is expected to have developed an ovarian/pelvic lesion first and **b** the other an omental lesion first. The grey and black horizontal lines denote the CA125 (0.015 cm^3^) and US detection limits (0.5 cm^3^). In both cases, the secondary site is expected to have started growing before the primary lesion reaches the US detection threshold. However, CA125 based detection provides a theoretical WOO of around 3 months (grey rectangle) for the case with the ovarian primary (**a**).

We estimated a median TTM of 13.1 months (IQR 5.6–21) between the initiation of disease in the primary and secondary sites for the 11 cases with growing lesions in both disease sites; this represents the most optimistic WOO for screening and suggests that five of these eleven cancers could not be caught by annual screening even at a detection resolution of one cell. Moreover, the secondary disease is expected to have initiated before the primary reaches the CA125/US detection thresholds for 3 out of 11, and 5 out of 11 cases respectively (Fig. 3b). Out of the eight cases that do reach the lower of the two thresholds before metastasis (Fig. 3a), six had a WOO of less than 6.5 months.

### 27% of all tumours in a virtual population metastasise before possible screen detection

Our simulations of a larger population yielded similar conclusions, with 72.8% and 55.3% of the virtual patients expected to reach the CA125 and US detection limits before metastasis. The median WOO for these individuals was 4.2 months (IQR 2.1–8.3) and 1.7 months (IQR 0.8–3.5) using CA125 and US based screening respectively.

## Discussion

Cancer growth kinetics dictate the probable success of early detection and clinical interventions. In this study, we used longitudinal clinical data from 34 HGSOC patients to understand how fast aggressive ovarian cancer tumours grow in the ovaries/pelvis and the omentum, how soon after tumour initiation they spread, and what the likely interval is for screening. We found that lesions in the ovaries/pelvis and the omentum doubled in volume every 2.2 months and 1.8 months while the median TTM for the 11 cases with growing lesions in both sites was 13.1 months. Our simulations suggest that although 72.8% of tumours could be caught before metastasis using CA125 based screening, they offer a median WOO of only 4.2 months for early detection. US based screening is predicted to carry even lower potential for early detection with only 55.3% of the cases reaching the detection limit before metastasis, with a median WOO of 1.7 months. These findings not only corroborate evidence of the inefficacy of annual screening, they also suggest that no practically feasible screening programme is likely to catch cancers before metastasis, given current thresholds of detection.

Our reported values for the kinetic parameters are consistent with those obtained using other approaches. The reported median TVDT for ovarian lesions falls within the ranges reported by Brown and Palmer [8] (2.5 months), Botesteneau et al. [11] (1.73 months), and Bedia et al. [12] (2.9 months). Similarly, the TTM of 13.1 months between the initiation of the ovarian/pelvic and omental lesions compares well with the period of 12.9 months [10] spent in stage I/II as estimated by a natural history model of aggressive (type-II) cancers. We find it encouraging that studies based on different types of data and different models arrive at similar estimates of the growth kinetics of HGSOC. However, our values for the TTM and WOO cannot be directly compared to the preclinical detectable periods (PCDP) of 11.9 months reported by Bedia et al., 2 years reported by Ishizawa et al. [29] or 2.9 years reported by Botesteanu et al. [11]. These values represent the interval between the earliest possible screen detection and clinical diagnosis. In contrast, the TTM represents the interval between lesion initiation at two different sites, while the WOO is the interval between possible screen detection and the *onset* of metastasis, which could occur earlier than the presentation of clinical symptoms.

We used two different modelling approaches for this work; the exponential model fitted to individual patient data enabled us to report the observed TVDT for the OV04 patient cohort without any extrapolation while the population-level Gompertz model was better equipped for backprojecting the course of disease and estimating the likely WOO. Our sensitivity analyses provide confidence that the reported population-level WOO will qualitatively hold under measurement errors and different choices of carrying capacity. The analyses also show that population-level Gompertz growth parameters are robust to observation errors even if individual estimates are susceptible to such perturbations.

The results of our work have important caveats. We use a small sample size of 34 patients from a single clinical site and empirical growth models for estimating the growth kinetics of HGSOC. The patient characteristics of these 34 cases were roughly representative of the overall dataset of 597 patients. However, the larger dataset itself is relatively ethnically homogeneous and contains little information on *BRCA* mutations of significance. We urge caution when extrapolating these findings to a more heterogeneous and global context despite the congruency of our estimated parameters with work done on a larger cohort [12]. We obtained the time since initiation of ovarian/pelvic and omental lesions by back-projecting a Gompertz model that has previously been shown to capture tumour growth in preclinical and clinical settings. Nevertheless, there is no clinical evidence that HGSOC follows a Gompertzian growth in-vivo. Moreover, our reliance on just two measurements per individual means that any model can fit the data; there is no way to evaluate different models by validating the back projected volumes against additional measurements done in preclinical studies [19, 30]. The limitation of having insufficient data is driven by a contradiction inherent to fast growing cancers – the speed of growth and dissemination that underpins their lethality also renders them difficult to observe and characterise. Numerical experiments suggest that faster growing tumours need fewer measurements to characterise their growth. However, we would need at least 3–4 measurements to accurately capture individual Gompertzian kinetics in the presence of even 5% measurement error [31]. Finally, our growth estimates do not account for non-growers or slow growers given that we impose a cut-off of 10% growth to negate the influence of artefacts.

The above limitations notwithstanding, this is the first study that uses (i) longitudinal volumetric data to infer the growth rates of HGSOC in different sites, and (ii) growth models of both primary and metastatic disease to estimate the WOO for screening. Notably, the kinetic parameters and study conclusion that we obtained through volume-based mechanistic modelling are consistent with those using a natural history model and biomarker-based kinetics. Our work provides further evidence of the challenges involved in ovarian cancer screening; it is likely that HGSOC grows too fast and spreads too soon for any meaningful screening-led reduction in mortality.

## Supplementary information

Supplemental File

Data Set 1

## Data Availability

The codes, raw data and fitted models are available at https://github.com/CCGE-Cambridge/HGSOC-GROWTH-KINETICS.

## Code availability

All the codes and raw data used in this study are available at a public github repository (See Data Access).

## References

[1] NHS England. Case-mix adjusted percentage of cancers diagnosed at stages 1 and 2 in England. 2021. https://digital.nhs.uk/data-and-information/publications/statistical/case-mix-adjusted-percentage-of-cancersdiagnosed-at-stages-1-and-2-in-england.

[2] NHS England. Cancer Survival in England, cancers diagnosed 2016 to 2020, followed up to 2021. 2023. https://digital.nhs.uk/data-and-information/publications/statistical/cancer-survival-in-england/cancersdiagnosed-2016-to-2020-followed-up-to-2021.

[3] Gaitskell K, Hermon C, Barnes I, Pirie K, Floud S, Green J, et al. Ovarian cancer survival by stage, histotype, and pre-diagnostic lifestyle factors, in the prospective UK Million Women Study. Cancer Epidemiol. 2022;76:102074. 34942490 10.1016/j.canep.2021.102074 PMC8785125

[4] Labidi-Galy SI, Papp E, Hallberg D, Niknafs N, Adleff V, Noe M, et al. High grade serous ovarian carcinomas originate in the fallopian tube. Nat Commun. 2017;8:1093. 29061967 10.1038/s41467-017-00962-1 PMC5653668

[5] Lisio MA, Fu L, Goyeneche A, Gao ZH, Telleria C. High-grade serous ovarian cancer: basic sciences, clinical and therapeutic standpoints. Int J Mol Sci. 2019;20:952. 30813239 10.3390/ijms20040952 PMC6412907

[6] Menon U, Gentry-Maharaj A, Burnell M, Ryan A, Kalsi JK, Singh N, et al. Mortality impact, risks, and benefits of general population screening for ovarian cancer: the UKCTOCS randomised controlled trial. Health Technol Assess. 2023;11:1–81. 10.3310/BHBR5832 PMC10542866 37183782

[7] Pinsky PF, Yu K, Kramer BS, Black A, Buys SS, Partridge E, et al. Extended mortality results for ovarian cancer screening in the PLCO trial with median 15 years follow-up. Gynecol Oncol. 2016;143:270–5. 27615399 10.1016/j.ygyno.2016.08.334 PMC5077651

[8] Brown PO, Palmer C. The preclinical natural history of serous ovarian cancer: defining the target for early detection. PLoS Med. 2009;6:e1000114. 19636370 10.1371/journal.pmed.1000114 PMC2711307

[9] Danesh K, Durrett R, Havrilesky LJ, Myers E. A branching process model of ovarian cancer. J Theor Biol. 2012;314:10–5. 22959913 10.1016/j.jtbi.2012.08.025 PMC3478401

[10] Havrilesky LJ, Sanders GD, Kulasingam S, Chino JP, Berchuck A, Marks JR, et al. Development of an ovarian cancer screening decision model that incorporates disease heterogeneity. Cancer. 2011;117:545–53. 21254049 10.1002/cncr.25624

[11] Botesteanu DA, Lee JM, Levy D. Modeling the dynamics of high-grade serous ovarian cancer progression for transvaginal ultrasound-based screening and early detection. PLoS One. 2016;11:e0156661. 27257824 10.1371/journal.pone.0156661 PMC4892570

[12] Bedia JS, Ian Jacobs HJ, Ryan A, Gentry-Maharaj A, Burnell M, Manchanda R, et al. Estimating the ovarian cancer CA-125 preclinical detectable phase, in-vivo tumour doubling time, and window for detection in early stage: an exploratory analysis of UKCTOCS. EBioMedicine 2025;112:105554. 10.1016/j.ebiom.2024.105554 PMC11782890 39808947

[13] Crispin-Ortuzar M, Woitek R, Reinius MAV, Moore E, Beer L, Bura V, et al. Integrated radiogenomics models predict response to neoadjuvant chemotherapy in high grade serous ovarian cancer. Nat Commun. 2023;14:6756. 37875466 10.1038/s41467-023-41820-7 PMC10598212

[14] Parkinson CA, Gale D, Piskorz AM, Biggs H, Hodgkin C, Addley H, et al. Exploratory analysis of TP53 mutations in circulating tumour DNA as biomarkers of treatment response for patients with relapsed high-grade serous ovarian carcinoma: a retrospective study. PLoS Med. 2016;13:e1002198. 27997533 10.1371/journal.pmed.1002198 PMC5172526

[15] Buddenkotte T, Rundo L, Woitek R, Escudero Sanchez L, Beer L, Crispin-Ortuzar M, et al. Deep learning-based segmentation of multi-site disease in ovarian cancer. Eur Radio Exp. 2023;7:77. 10.1186/s41747-023-00388-z PMC10700248 38057616

[16] Steel GG, Lamerton LF. The growth rate of human tumours. Br J Cancer. 1966;20:74. 5327764 10.1038/bjc.1966.9 PMC2008056

[17] Spratt JA, von Fournier D, Spratt JS, Weber EE. Decelerating growth and human breast cancer. Cancer. 1993;71:2013–9. 8443753 10.1002/1097-0142(19930315)71:6<2013::aid-cncr2820710615>3.0.co;2-v

[18] Laird AK. Dynamics of tumor growth. Br J Cancer. 1964;18:2948–61. 10.1038/bjc.1964.55 PMC2071101 14219541

[19] Benzekry S, Lamont C, Beheshti A, Tracz A, Ebos JML, Hlatky L, et al. Classical mathematical models for description and prediction of experimental tumor growth. PLoS Comput Biol. 2014;10:e1003800. 25167199 10.1371/journal.pcbi.1003800 PMC4148196

[20] Winsor CP. The Gompertz curve as a growth curve. Proc Natl Acad Sci. 1932;18:1–8. 16577417 10.1073/pnas.18.1.1 PMC1076153

[21] Norton L. A Gompertzian model of human breast cancer growth. Cancer Res. 1988;48:7067–71. 3191483

[22] Kuhn E, Lavielle M. Maximum likelihood estimation in nonlinear mixed effects models. Comput Stat Data Anal. 2005;49:1020–38.

[23] The MathWorks Inc. MATLAB version: 24.1.0 (R2024a) Update 3. 2024.

[24] Saida T, Tanaka YO, Matsumoto K, Satoh T, Yoshikawa H, Minami M. Revised FIGO staging system for cancer of the ovary, fallopian tube, and peritoneum: important implications for radiologists. Jpn J Radio. 2016;34:117–24. 10.1007/s11604-015-0513-3 26696400

[25] Prakash P, Cronin CG, Blake MA. Role of PET/CT in ovarian cancer. Am J Roentgenol. 2010;194:20210117. 10.2214/AJR.09.3843 20489063

[26] Healy JC. Detection of peritoneal metastases. Cancer Imaging. 2001;1:4–12. 18203670 10.1102/1470-7330.2001.002 PMC4448957

[27] Mathieu KB, Bedi DG, Thrower SL, Qayyum A, Bast RC. Screening for ovarian cancer: imaging challenges and opportunities for improvement. Ultrasound Obstet Gynecol. 2018;51:293–303. 28639753 10.1002/uog.17557 PMC5788737

[28] Lutz AM, Willmann JK, Cochran FV, Ray P, Gambhir SS. Cancer screening: a mathematical model relating secreted blood biomarker levels to tumor sizes. PLoS Med. 2008;5:1287–97. 10.1371/journal.pmed.0050170 PMC2517618 18715113

[29] Ishizawa S, Niu J, Tammemagi MC, Irajizad E, Shen Y, Lu KH, et al. Estimating sojourn time and sensitivity of screening for ovarian cancer using a Bayesian framework. JNCI J Natl Cancer Inst. 2024;116:1798–806. 39038822 10.1093/jnci/djae145 PMC13396154

[30] Vaghi C, Rodallec A, Fanciullino R, Ciccolini J, Mochel JP, Mastri M, et al. Population modeling of tumor growth curves and the reduced Gompertz model improve prediction of the age of experimental tumors. PLoS Comput Biol. 2020;16:e1007178. 32097421 10.1371/journal.pcbi.1007178 PMC7059968

[31] Harshe I, Enderling H, Brady-Nicholls R. Predicting patient-specific tumor dynamics: how many measurements are necessary? Cancers. 2023;15:1368. 36900161 10.3390/cancers15051368 PMC10000065

