# Supplementary material for: Growth kinetics of high-grade serous ovarian cancer: implications for early detection
Source: Br J Cancer. 2025 Jun 12;133(4):533–8. doi: 10.1038/s41416-025-03082-6 (PMC12356848; doi:10.1038/s41416-025-03082-6)
Supplement: Supplementary file 1 — Supplemental File [file 41416_2025_3082_MOESM1_ESM.docx]

Supplemental material

# Note 1: Deriving the confidence interval of $t_{1}^{pop}$

Letting $q=\beta t_{1}$, we first calculate the population level $t_{1}^{pop}$ which is the geometric mean of the log normal distribution of $\ln t_{1}$.

$$\left( \ln t_{1} \right)^{pop}=\left( \ln q \right)^{pop}-\left( \ln\beta\right)^{pop}$$

$$t_{1}^{pop}=\exp\left( \ln t_{1} \right)^{pop}$$

For the confidence interval (CI) of $t_{1}^{pop}$ we start by providing the formula for the standard error of the difference between two independent, normally distributed variables:

$$SE_{X-Y}=\sqrt{SE_{X}^{2}+SE_{Y}^{2}}$$

Here, $X-Y=\left( \ln t_{1} \right)^{pop}$, $X=\left( \ln q \right)^{pop}$ and $Y=\left( \ln\beta\right)^{pop}$

$$SE_{\left( \ln t_{1} \right)^{pop}}=\sqrt{SE_{\left( \ln q \right)^{pop}}^{2}+SE_{\left( \ln\beta\right)^{pop}}^{2}}$$

Now, the confidence interval of $\left( \ln t_{1} \right)^{pop}$ is

$$CI_{\left( \ln t_{1} \right)^{pop}}=\left[ \left( \ln t_{1} \right)^{pop}\mp t_{.975}\cdot SE_{\left( \ln t_{1} \right)^{pop}} \right]$$

We use the t-distribution as we only have 24 individuals for the ovarian lesions ($t_{.975}=2.069$) and 21 individuals for the omental lesions ($t_{.975}=2.086)$.

Finally, we estimate the CI for the population level estimate of $t_{1}$:

$$CI_{t_{1}^{pop}}=\exp CI_{\left( \ln t_{1} \right)^{pop}}$$

Note that we seek the CI estimate for the geometric mean, $\exp\left( \ln t_{1} \right)^{pop}$, and not the CI for the mean of $t_{1}$, $\mu_{t_{1}}=\exp(\mu_{\ln t_{1}}+\sigma_{\ln t_{1}}^{2})$. This is why we use the naïve approach to CI estimation here.

# Note 2: Ovarian and omental volumes from the 34 cases

Ovarian (Figure S.1a) and omental (Figure S.1b) volumes from the 34 cases used in this study. The blue lines represent the 11 cases with growing lesions in both disease sites, while the black lines denote the cases with growing ovarian (13) or omental (10) lesions only. Cases coloured red are those that were discarded for volume calculation due to a lack of growth (<10%), or their extremely small size (<0.05cm^3^). The y axis is in log-scale to better distinguish the different magnitudes of volumes.


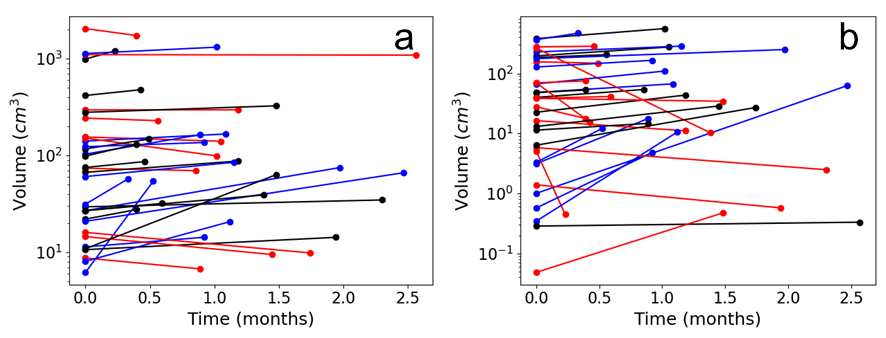


***Figure S.1:*** *Raw* ***(a)*** *ovarian and* ***(b)*** *omental volumes from the 34 individuals with 2 scans before treatment.*

# Note 3: Sensitivity of model estimates to errors in measurement and choice of maximal disease burden

We reported our results assuming that the segmented volumes represent the ground truth. However, measurement error is inherent in segmentation and volume calculation. We studied the sensitivity of the estimated population mean, $t_{1}^{pop}$, to these errors by applying 10% random Gaussian noise to the volumes before fitting the model. We required that there should be at least 10% volume change between the two measurements after applying the noise; this ensured consistency with the actual analysis. We repeated this 20 times and calculated the coefficient of variation (CV) of $t_{1}^{pop}$ across these runs.

| $\hat{V_{j}^{i}}=V_{j}^{i}\left( 1+e_{j}^{i} \right)$ $e_{j}^{i}\sim N(0,0.1)$ |  |
| --- | --- |

The estimated population level $t_{1}^{pop}$ had a coefficient of variation (CV) of 5.3% and 9.8% for ovarian and omental lesions respectively across the 20 runs with noisy measurements. However, the distributions themselves were visibly different (Figures S.2a and S.2b), suggesting that estimates of the population mean are robust to measurement errors even when individual estimates can be different. The median root mean squared errors across the runs was 3.5e-15 (IQR: 1.4e-15 – 7.7e-15) and 0.017 (IQR: 1e-14 – 0.036) for ovarian and omental measurement sensitivity respectively.

We followed a similar approach to understand how our results were impacted by the choice of the maximal carrying capacity of the ovarian and omental lesions. We varied the $V_{\infty}$ from 2,000 to 10,000 cm^3^ for each disease site and compared the fitted distribution of $t_{1}$ for each case. We found that the estimated $t_{1}^{pop}$ had a CV of 7.9% and 9.4% when the $V_{\infty}$ was varied between 2000 and 10000 cm^3^ in the ovarian (Figure S.2c) and omental (Figure S.2d) sites respectively. This variation dropped to 3.1% and 4.6% for carrying capacities at or above the values set for the ovarian (5000cm^3^) and omental (3000cm^3^) lesions respectively, indicating a negligible impact of the choice of $V_{\infty}$ on our findings.


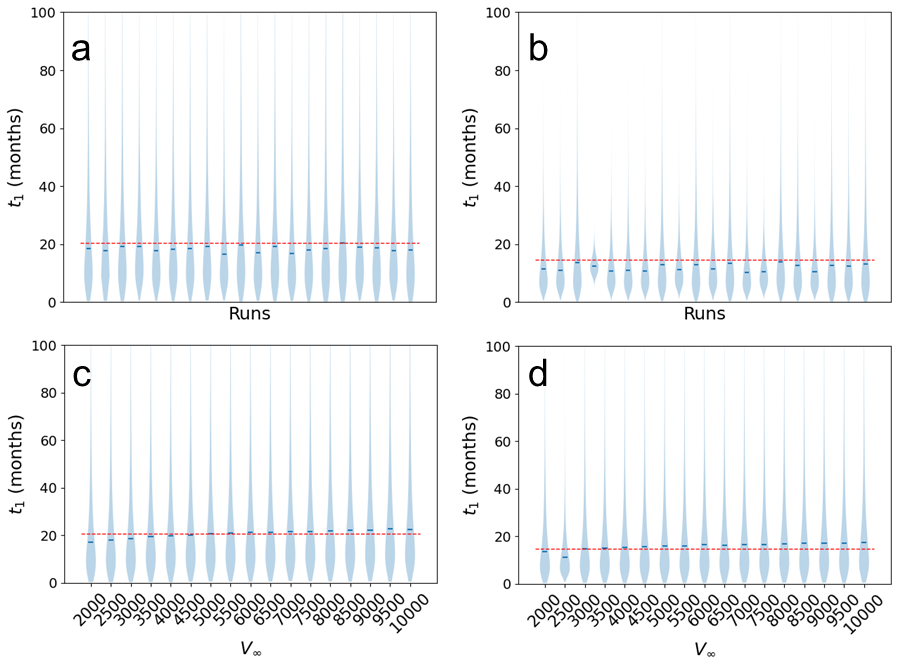


***Figure S.2:*** *Sensitivity of the t1 estimates to measurement errors and the choice of* $V_{\infty}$*. The violin plots in the top row show the log-normal distributions of* $t_{1}$ *estimates for each of the 20 runs with 10% Gaussian noise. We observed a CV of 5.3% and 9.8% in the estimated population-level* $t_{1}^{pop}$*(blue horizontal lines) for* ***(a)*** *ovarian and* ***(b)*** *omental lesions respectively. The red dashed lines represent the estimated* $t_{1}^{pop}$ *without any noise. The bottom row shows the moderate rise in the estimated* $t_{1}^{pop}$ *as the assigned* $V_{\infty}$ *for* ***(c)*** *ovarian and* ***(d)*** *omental lesions is increased from 2000 cm3 to 10,000 cm3. The red dashed line here corresponds to the value of* $t_{1}^{pop}$*estimated using* $V_{\infty}$ *values used for the analysis in the paper - 5000cm^3^ for ovarian lesions and 3000cm^3^ for omental lesions. The CV in* $t_{1}^{pop}$*was 7.9% and 9.4% for ovarian and omental sites respectively.*
